# Development and initial cognitive testing of the Digital Equity Screening Tool (DEST): Community participatory approach to assessing digital inequality

**DOI:** 10.1017/cts.2022.451

**Published:** 2022-08-25

**Authors:** Pravesh Sharma, Christi A. Patten, Jon C. Tilburt, Andrea L. Cheville, Joshua C. Pritchett, LaPrincess C. Brewer, Richard O. White, Sydney S. Kelpin, Monica Albertie, Tabetha A. Brockman, Chyke A. Doubeni

**Affiliations:** 1 Behavioral Health Research Program, Mayo Clinic, Rochester, MN, USA; 2 Department of Psychiatry and Psychology, Mayo Clinic Health System, Eau Claire, WI, USA; 3 Department of Psychiatry and Psychology, Mayo Clinic, Rochester, MN, USA; 4 Center for Clinical and Translational Science, Mayo Clinic, Rochester, MN, USA; 5 Biomedical Ethics Research Program, Mayo Clinic, Scottsdale, AZ, USA; 6 Department of Physical Medicine and Rehabilitation, Mayo Clinic, Rochester, MN, USA; 7 Division of Hematology, Mayo Clinic, Rochester, MN, USA; 8 Division of Medical Oncology, Mayo Clinic, Rochester, MN, USA; 9 Cardiovascular Rehabilitation Program, Department of Cardiovascular Diseases, Mayo Clinic, Rochester, MN, USA; 10 Center for Health Equity and Community Engagement Research, Mayo Clinic, Jacksonville, FL, USA; 11 Division of Community Internal Medicine, Mayo Clinic, Rochester, MN, USA; 12 Department of Family Medicine, Mayo Clinic, Rochester, MN, USA

**Keywords:** Digital access and literacy, COVID-19, community engagement, community engagement studio, community advisory board

## Abstract

COVID-19 has widened the existing digital divide, especially for people from socially and economically deprived communities. We describe a program evaluation using a community participatory approach to develop self-reported items of patient experience with technology inclusive of digital access and literacy. The feedback received from Community Advisory Boards and Community Engagement Studio members led to the evaluation and refinement of the individual items. The community-based participatory approach highlighted in our paper to develop these items could serve as a model for other screening tool development for enhancing equity and inclusiveness in clinical care and research.

## Introduction

The COVID-19 pandemic led to an acceleration in the use of digital health services in the United States with the potential to widen existing disparities in access to healthcare for people from groups that are socially and economically disadvantaged, including rural communities [1−3]. Digital health inequities may result from structural barriers such as lack of broadband internet connection, smart device, limited digital literacy (ease or comfort with using technology), and language or cultural barriers [3−5]. For those reasons, Digital Equity frameworks seek to incorporate digital access and literacy as social determinants of health (SDoH) [4]. A person’s ability to use digital tools has emerged as a critical determinant of the quality of health care received, yet clinicians lack accessible and standardized digital health assessment tools. Existing tools assess mobile phone adoption and internet access [6] and digital literacy [7] and the use of patient electronic health record messaging systems (or patient portal) has also been used as a proxy for digital access and fluency/facility [1,8]. Census tract-level data can also determine residential broadband connections [9]. However, few measures assess *patients’ self-reported experience* of accessing and interacting with technology for health care delivery [4]. The design of such measures should involve patient and community stakeholders (i.e., end-user feedback) [4,10− 12]. We used a community-engaged approach to develop items to assess digital access and literacy for clinical and research applications to address the current paucity of patient-reported assessment tools. Our eventual goal is to integrate such items into SDoH modules in the EHR. Devising and refining such a tool with an inclusive person-centered design approach could optimize the ability to address digital inequities [12,13].

## Methods

This project was deemed exempt from the Mayo Clinic Institutional Review Board.

### Study Design and Setting

This project was conducted across all campuses of the Mayo Clinic (Rochester, MN; Jacksonville, FL; and Phoenix, AZ) and the Mayo Clinic Health System (MCHS) with the involvement of community members drawn from community advisory boards (CAB) and Community Engagement (CE) Studio. As described elsewhere, Mayo Clinic has advisory boards at each campus that provide guidance on community-based research and overall research strategy for research programs and centers, including the Center for Clinical and Translational Science (CCaTS) and Center for Health Equity and Community Engagement Research (CHCR). CE Studios are a platform for community engagement that includes facilitated discussions to obtain meaningful feedback on a topic of interest from community members, patients, or other stakeholders with lived experience with researchers’ population of interest who are deemed experts [14].

### Item Development Process

Our project team consisted of an expert panel of rural health and health disparities researchers and clinicians, and patient advocates from the Mayo Clinic enterprise. The panel compiled the preliminary items based on empirical research, existing literature [15], and the panel’s collective research and clinical expertise with diverse communities. During this stage a stepwise consensus process strategy [16] was used by the panel to eliminate the items that were less useful and develop an initial set of four self-reported items (called V1) that covered four major constructs based on our objectives: *access to smart devices*, *high-speed internet access*, *comfort with using technology*, and *need for assistance to use technology* (See Table 1).

**Table 1.**
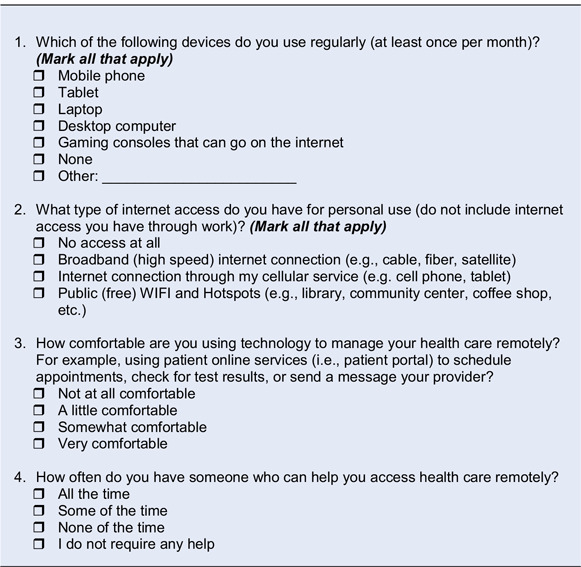
Original digital screening tool (V1)

A fifth item related to language barriers was also devised as part of the group’s process (See Table 2). The next step was to seek feedback about items for their relevance and importance from community members participating in CAB and CE Studios.

**Table 2.**
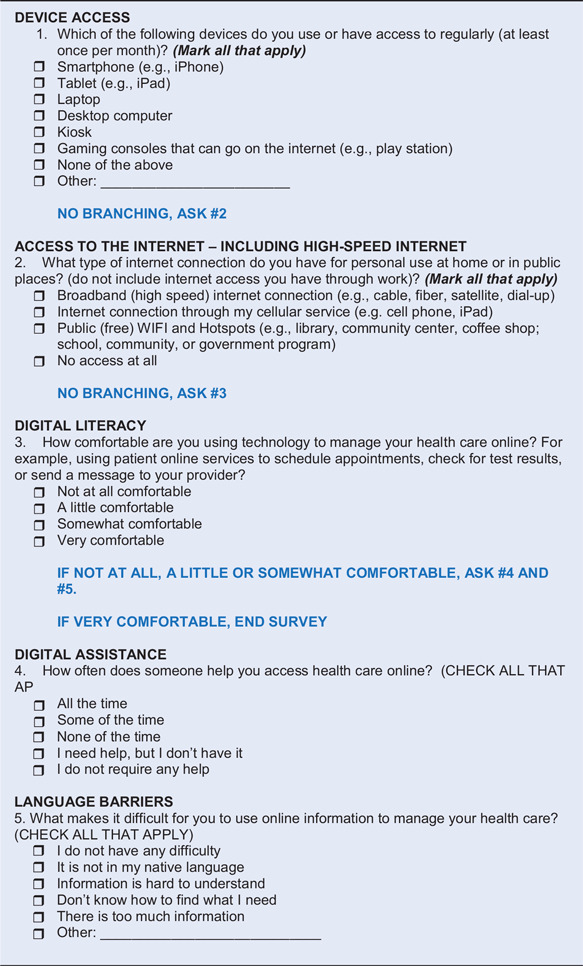
Revised version (V2): Digital Equity Screening Tool (DEST)

### Patient and Community Member Feedback

The set of four items (V1) (Table 1) was evaluated for content validity through the guidance and advice received in three separate CABs, and a CE Studio [17,18].

#### Community Advisory Boards (CABs).

The CAB staff provided members an electronic version of the V1 prior to the virtual meeting. During the meeting two study staff (PS and CP) presented and facilitated the discussion at each site in November and December of 2021. Participants were 10 CAB members in the Midwest, 20 in Arizona, and 16 in Florida. Individuals from diverse racial and ethnic backgrounds, gender identities, and both younger and older participants were represented.

We obtained structured cognitive interview feedback on all four items (V1) through discussions and group interactions among CAB members [17,18]. During the discussion each question was displayed, and members provided feedback about the questions flow, understandability, relevance, and question content [16,18]. Next, the study staff shared the fifth item on language and asked about adding it in the context of respondent burden, wording, and response options. Members also had the opportunity to provide open-ended feedback. Participants received a $25 to $50 honorarium based on the respective CAB’s precedent. The CAB coordinators took detail notes.

#### Community Engagement [16] Studio

Participants were invited by program staff from all three Mayo Clinic sites by phone, text message, and email. The 90-minute CE Studio was held virtually in December 2021. The ten individuals who attended included five Mayo Clinic patients (Midwest *n* = 2, Arizona *n* = 2, and Florida *n* = 1) and five patients at local community clinics (Midwest *n* = 2, Arizona *n* = 1; Florida *n* = 2). During the CE Studio, two program staff (PS and CP) provided an overview of the project. A trained program staff member facilitated the discussion, and another took detail notes. Similar to the CABs, during the discussion each question was displayed, and participants provided feedback about the questions flow, understandability, relevance, and question content [17,18]. Participants received an honorarium of $40 gift card each as a thank you for their time.

### Evaluation

An iterative, inductive, content analysis [19] approach was used to generate themes from the detailed notes gathered across the CABs and CE Studio. Two of the authors jointly coded the responses (PS, CP) and a third author (TB) resolved any disagreements until a consensus was reached [20,21,22].

## Results

### Overall Feedback Themes

A major theme from the community-based feedback was the perceived importance of the items’ underlying objective – enhancing clinical care access and use. The participants discussed possible strategies of linking items to other resources such as patient digital education through a digital navigator [23].

A great deal of discussion focused on the format of items administration because patients may not have digital device/internet access to answer the items and their overall digital health literacy may present as a possible barrier. It was, therefore, recommended the items be administered in person and by someone who could read the items to the patient. The survey length was thought to be appropriate with no changes recommended. It was recommended the items be available in other languages.

### Item-specific Feedback

#### Item 1: device access.

Participants recommended changes in the wording and added examples to enhance comprehension, particularly for those who may have access to a device but not own one, so the item stem might read “…devices do you use or have access to regularly…?” Response option recommendations included changing “mobile phone” to smartphone or cell phone, providing examples for “tablet” (i.e., iPad) and “gaming consoles” (i.e., play station), and adding “kiosk” as an option.

#### Item 2: internet access.

Participants recommended clarifying access versus use and locations of use. Not everyone may be clear on the term “internet access.” The patient may participate in an appointment from the parking lot of a grocery store—and not always from home. Thus, participants suggested rephrasing the item stem to “What type of internet connection do you have for personal use at home or in public locations?” For response options, it was recommended to add choices for “dial-up” internet connection and internet services through government, school, or community programs. Participants suggested the response option “no access at all” be moved to be presented as the final choice.

#### Item 3: digital literacy.

Separate from device access, some people may not feel comfortable using technology. Jargon like “patient portal” and “remotely” did not resonate, whereas the term “online” seemed appropriate to respondents. Because creating online accounts and remembering passwords are key skills, language referring to “creating an account” was added to the literacy item stem.

#### Item 4: digital assistance.

As with item 3, participants suggested replacing “remotely” with “online” in the item stem. Minor simplification of language was proposed changing the stem from “How often do you have someone who can help you access health care remotely?” to “How often does someone help you access health care online?” And adding a (Mark all that apply) response option and expanding choices to include “I’d like help, but I don't have any” or “I need help, but I don't have it” along with “I do not require any help.”

#### Language barriers (possible item 5).

Item 5 was the result of direct participant feedback. Because phrases like “digital information” and “health-related decisions” were not clear they recommended, “What makes it difficult for you to use online information to manage your health care?” And an “other: specify_____” response choice was suggested.

### Revised Items

The response themes were discussed among the panel and resulted in a revised version (V2), named the Digital Equity Screening Tool (DEST) with five items and a proposed branching logic as shown in Table 2.

### Dissemination

We are in the process of sharing our results with each CAB during one of their regularly scheduled meetings. We will mail results to CE Studio participants via a newsletter.

## Discussion

In this program evaluation, we used a community participatory approach to develop new self-reported items of patient experience with technology comprehensive of digital access and literacy. The feedback we received from participants shaped not only the specific items of the DEST but the format of its administration. Participants had several areas of feedback to enhance the understandability of items.

Our approach has limitations, such as we asked for feedback during patient and community meetings held using a virtual format which may lack representation of those without digital access, comfort, or skills. Therefore, the next steps in item development would be to administer the DEST in person with a group of patients who are underresourced and evaluate if the content validity of the items has improved. Another limitation is that community members were drawn from existing advisory boards who have worked previously with our institution and thus may have more advanced knowledge of healthcare and research, and we obtained feedback from a small group of patients, who may not be representative of the community and patient populations the DEST is intended to identify. Therefore, we are in the process of testing DEST in a larger and more diverse sample of patients and community members. Nonetheless, the inclusive design of the DEST development and cognitive testing process is a strength of this program evaluation and a vital first step toward addressing inequities in digital healthcare.

It is important to note that the approach highlighted in this paper is only the first of many future steps required to validate the DEST. The final items and item response [24] scales of the DEST will be selected based on rigorous psychometric methods in representative samples. For example, we plan to evaluate the relationships of patient experience (DEST items) with objective measures such as AI-generated algorithms for household and neighborhood area access to broadband internet [25]. Ultimately, we envision the DEST would be included within the SDoH questions asked at clinic visits and available within the EHR.

The DEST has potential research applications as well such as enhancing inclusiveness in the next generation of decentralized and digital late-stage translational science including clinical trials. Trials being increasingly conducted via digital means will require access to devices and internet connections along with comfort with the use of technology. Providing a loaner tablet with data plan remuneration for the study duration would be one possible solution for individuals with digital access barriers. The DEST could also be used to monitor digital access on a population level, including assessing disparities and changes over time, particularly to assess whether specific sub-populations are falling further behind in digital healthcare delivery. And the community participatory approach we deployed to develop the DEST could serve as a model for other screening tool development for enhancing equity and inclusiveness in clinical care and research.
